# Estimating hydrogen absorption energy on different metal hydrides using Gaussian process regression approach

**DOI:** 10.1038/s41598-022-26522-2

**Published:** 2022-12-19

**Authors:** Majedeh Gheytanzadeh, Fatemeh Rajabhasani, Alireza Baghban, Sajjad Habibzadeh, Otman Abida, Amin Esmaeili, Muhammad Tajammal Munir

**Affiliations:** 1 Surface Reaction and Clean Energy Materials Laboratory, Chemical Engineering Department, Amirkabir University of Technology (Tehran Polytechnic), Tehran, Iran; 2 Chemical Engineering Department, Fouman Faculty of Engineering, University of Tehran, Fouman, Iran; 3 Chemical Engineering Department, Amirkabir University of Technology (Tehran Polytechnic), Mahshahr Campus, Mahshahr, Iran; 4 College of Engineering and Technology, American University of the Middle East, 54200 Egaila, Kuwait; 5 Department of Chemical Engineering, School of Engineering Technology and Industrial Trades, College of the North Atlantic - Qatar, Doha, Qatar

**Keywords:** Chemistry, Energy science and technology, Engineering, Materials science

## Abstract

Hydrogen is a promising alternative energy source due to its significantly high energy density. Also, hydrogen can be transformed into electricity in energy systems such as fuel cells. The transition toward hydrogen-consuming applications requires a hydrogen storage method that comes with pack hydrogen with high density. Among diverse methods, absorbing hydrogen on host metal is applicable at room temperature and pressure, which does not provide any safety concerns. In this regard, AB_2_ metal hydride with potentially high hydrogen density is selected as an appropriate host. Machine learning techniques have been applied to establish a relationship on the effect of the chemical composition of these hosts on hydrogen storage. For this purpose, a data bank of 314 data point pairs was used. In this assessment, the different A-site and B-site elements were used as the input variables, while the hydrogen absorption energy resulted in the output. A robust Gaussian process regression (GPR) approach with four kernel functions is proposed to predict the hydrogen absorption energy based on the inputs. All the GPR models' performance was quite excellent; notably, GPR with Exponential kernel function showed the highest preciseness with R^2^, MRE, MSE, RMSE, and STD of 0.969, 2.291%, 3.909, 2.501, and 1.878, respectively. Additionally, the sensitivity of analysis indicated that ZR, Ti, and Cr are the most demining elements in this system.

## Introduction

Energy demand has increased exponentially in recent years, reaching over 18 TW. In the subsequent years, this market growth is expected to continue^1^. Nowadays, fossil fuels account for over 80% of global energy consumption^2–5^. However, due to the environmental issues, the transition to renewable energy sources is critical^6^. In this regard, hydrogen can revolutionize renewable energy systems as a fuel and a clean energy carrier. It could be the basis for establishing carbon-free fuels^7,8^. Hydrogen energy has been among the most popular energy sources in recent years. This is since it has a higher energy content and causes fewer environmental issues than fossil fuels^9^. Hydrogen has a far higher energy density of 142 Mj kg^−1^ than fossil fuels, with a density of 47 Mj kg^−1^^10^. It is estimated that about 35% of European vehicles will be hydrogen-powered by 2040^9^. In addition, hydrogen energy will provide around 34% of the world's energy demands by 2050^11^. Even though hydrogen is a prevalent element in nature, it is rarely found in pure form. As a result, several chemicals, electrochemical, photoelectrochemical, thermal, and microbiological approaches have been developed for producing it^12–14^. More than 50 million tons of hydrogen are produced annually in the world^15^.

Hydrogen may be stored in three primary ways, including gas, liquid, and solid-phase storage. Solid-phase storage is one of the most promising storage technologies owing to its ability to operate at room temperature and atmospheric pressure, as well as its excellent safety and low energy loss^16–21^. Metal hydrides have been noticed as a hydrogen storage material in solid-state conditions^22–27^ and are produced by absorption of hydrogen molecules on a metallic/intermetallic host^28^. The gravimetric density of hydrogen absorbed in these compounds is about 1–3 wt%^5,29^. Different metal hydrides have been identified and examined so far, including AB, AB_2_, AB_3_, AB_5_, and A_2_B, in which A and B are two types of metals or a group of metals. The AB_2_ metal hydride is the most promising type for hydrogen storage due to its easy activation, fast kinetics, and favorable pressure conditions^30^. In AB_2_ alloys, element A contains hydride constituent of elements such as Ti, Zr, Ta, and Hf, while element B contains transition metals such as Fe, Co, Ni, Mn, Cr, and V^31,32^. The C14 and C15 with a hexagonal and face-center-cubic structure, respectively, are the laves phases of the AB_2_ metal hydrides^33^.

Thus, the element selection for A-site and B-site of AB_2_ compounds influences their hydrogen storage performance. In order to investigate the effect of different elements or dopants on the hydrogen storage properties, traditional approaches, such as basic laws, computational modeling, and experimental investigations, are costly, time-consuming, and associated with numerous trials and errors, making them challenging and inefficient. Thus, to save time, energy, and cost, mainly when a complicated nonlinear relationship exists between the parameters and the performance, alternative machine learning (ML) techniques could be effective assessment methods.

The ML has become a prominent field of research and approach in developing and selecting advanced energy materials in recent years^34–40^. So far, various machine learning algorithms have employed hydrogen storage by metal hydride systems. For example, Griffin and Darsey estimated entropy, enthalpy, the temperature at 1 atm, pressure at 25 C, and the weight percent of hydrogen stored in metal hydrides using artificial neural networks. For the above parameters, the average correlation coefficient of R^2^ was 0.8888, 0.9561, 0.9381, 0.9935, and 0.9569, respectively^41^. To estimate the hydrogen storage capacity in metal hydrides, Rahnama et al. utilized four models: linear regression, neural network, Bayesian linear regression, and boosted decision tree. The R^2^ of the utilized models were 0.50, 0.60, 0.56, and 0.83, respectively, indicating that the boosted decision tree performed better than the other models^42^. In another study, Rahnama et al. classified metal hydrides using four classifiers: multiclass logistic regression, multiclass decision forest, multiclass decision jungle, and multiclass neural network. The accuracy of the used models was 0.47, 0.60, 0.62, and 0.80, respectively, indicating that the multiclass neural network classifier performed better than the other classifiers. This classification was based on the properties of metal hydrides, including the weight percentage of hydrogen, heat of formation, and operating temperature and pressure^43^. Suwarno et al. used their research to use multivariate regression, decision tree, and random forest models. The heat of formation, phase abundance, and hydrogen storage capacity of AB_2_ metal hydrides were all estimated using these models. The random forest model showed the most outstanding performance among the three models, with an average R^2^ value of 0.722^44^. Determining the pressure-composition-temperature (PCT) curve is an important issue in metal hydrides. This issue was considered in the research of Kim et al., where random forest (RF), K-nearest neighbor (KNN), and deep neural network (DNN) models were used. The deep neural network (DNN) model exhibited the greatest performance among the three models, with an average correlation coefficient R^2^ of 0.9307^45^.

In the present study, for the first time, the Gaussian process regression (GPR) model with four kernel functions was used to estimate the energy of hydrogen absorption (ΔH) on the surface of the hydride alloys. The elements of A and B in AB_2_ compounds were chosen as input variables to establish a relationship between the chemical composition of AB_2_ and hydrogen storage properties. For this purpose, a substantial experimental data bank was applied. The developed model was evaluated by several error and statistical parameters. Also, sensitivity analysis was performed to find the most determining elements in the hydrogen storage on metal hydrides.

## Methodology

### Data collection

A set of 314 pairs of AB_2_ alloys were collected and presented in the Supplementary Information from the literature^44^. They include the information of constituent elements and ΔH absorption (in KJ/(molH_2_)).

It is worth mentioning that, in the pressure-composition-temperature diagram, some of the ΔH of these alloy couples are tacitly explained but are not clearly stated in the publications. The van't Hoff Law, as shown in the following equation, was used to calculate the aforementioned ΔH.

1 $$ln{P}_{eq}=\frac{\Delta H}{RT}-\frac{\Delta S}{R}$$

In order to determine the equilibrium pressure, the computation was done by choosing a midpoint from the plateau of the pressure-composition graph. The temperature value in the pressure-composition phase diagram is constant because R, the universal gas constant, is used in the calculation. The term S is assumed to have a constant value of − 110 kJ/(mol H_2_ K).

**Figure 1:**
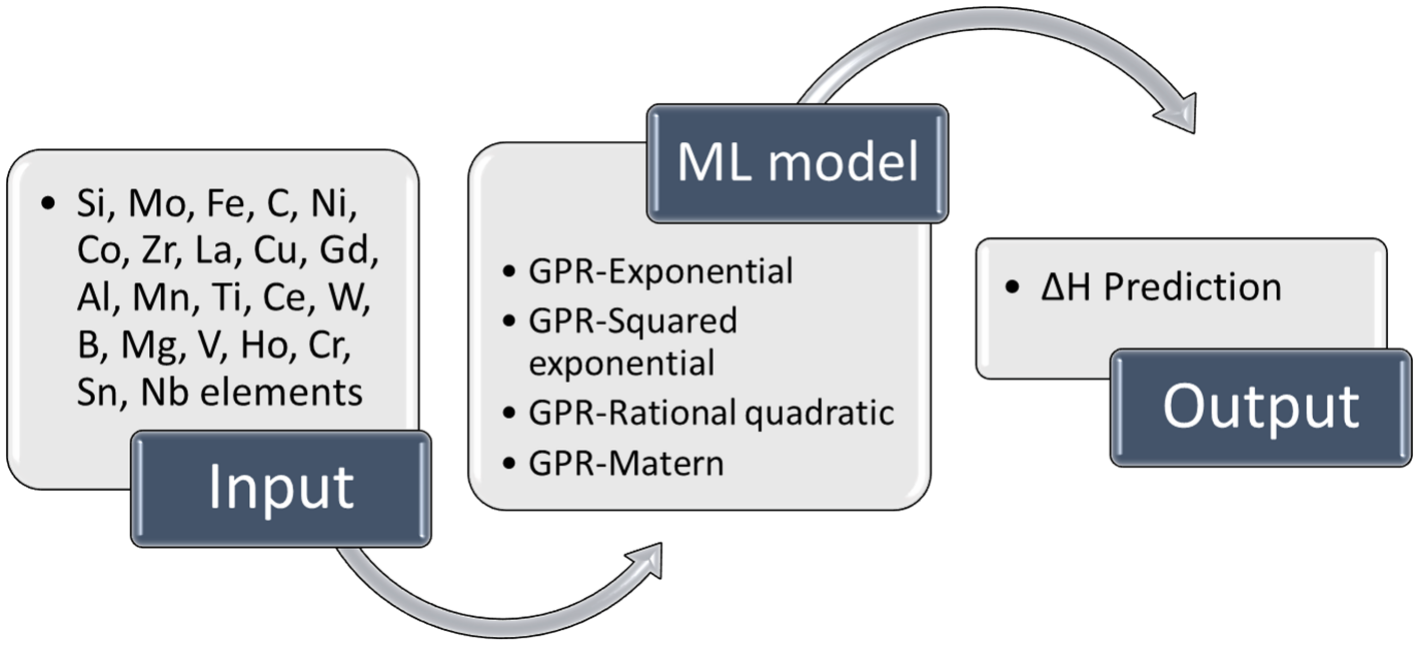
Steps and analysis parameters through the study.

As depicted in Fig. 1, 22 alloying elements of Si, Mo, Fe, C, Ni, Co, Zr, La, Cu, Gd, Al, Mn, Ti, Ce, W, B, Mg, V, Ho, Cr, Sn, and Nb are the input parameters while the ΔH is the output of the model to see the effect of each parameter on the hydrogen storage conditions. In this work, 70% of data was separated coincidentally as training data to develop the model, and the rest (30% data) was used as testing data for prediction to evaluate the model's accuracy. Several statistical factors were calculated to quantify the established model preciseness, including R^2^, standard deviation (STD), mean-square error (MSE), mean relative error (MRE), and root-mean-square error (RMSE). Consiering *y* and *x* as the predicted and experimental values respectively, these factors are defined as follows:

2 $$R^{2} = 1 - \frac{{\mathop \sum \nolimits_{i = 1}^{n} \left[ {y_{i} - x_{i} } \right]^{2} }}{{\mathop \sum \nolimits_{i = 1}^{n} \left[ {y_{i} - x_{m} } \right]^{2} }}$$

3 $$STD = \sqrt {\mathop \sum \limits_{i = 1}^{n} \frac{{\left( {y_{i} - x_{m} } \right)^{2} }}{n}}$$

4 $$MSE = \frac{1}{n}\mathop \sum \limits_{i = 1}^{n} \left( {y_{i} - x_{i} } \right)^{2}$$

5 $$RMSE = \sqrt {\frac{{\mathop \sum \nolimits_{i = 1}^{n} \left( {y_{i} - x_{i} } \right)^{2} }}{n}}$$

6 $$MRE = \frac{1}{n}\mathop \sum \limits_{i = 1}^{n} \frac{{\left| {y_{i} - x_{i} } \right|}}{{x_{i} }}$$

### Gaussian process regression

In comparison to support vector machines and artificial neural networks, Gaussian process regression with its super-parameters, which can be adaptively attained, is easy to perform. Also, the confidence interval (i.e., the uncertainty of the model prediction) can be obtained by this method^35,46^.

In GPR modeling $L = \left\{ {x_{L \cdot i} \cdot y_{L \cdot i} } \right\}_{i = 1}^{{n_{1} }}$ and $T = \left\{ {x_{T \cdot i} \cdot y_{T \cdot i} } \right\}_{i = 1}^{{n_{2} }}$ are arbitrarily selected training and testing data sets with input and output parameters of *x* and *y*, respectively. The modeling begins by:

7 $$y_{L.i} = f\left( {x_{L \cdot i} } \right) + \varepsilon_{L \cdot i} \quad \cdot \quad i = 1 \cdot 2 \cdot 3 \cdot \ldots \cdot n_{1}$$

8 $$\varepsilon \sim N\left( {0 \cdot \sigma_{noise}^{2} I_{n} } \right)$$

where $\varepsilon$, σ^2^_noise_, and *I*_*n*_ are the observation noise, the variance of the noise, and the unit array. Similar to the traing data, we have for the test data:

9 $$y_{T.i} = f\left( {x_{T.i} } \right) + \varepsilon_{T.i} \quad .\quad i = 1.2.3. \ldots.n_{2}$$

In GPR method, *f*(*x*) is a random function which defined by its corresponding covariance *k*(*x, x′*) (also called kernel) and mean *m*(*x*) functions.

10 $$f\left( {x_{L \cdot i} } \right) \sim GP\left( {m\left( x \right) \cdot k\left( {x \cdot x^{\prime} } \right)} \right)$$

Although *m*(*x*) can be obtained by applying explicit basis functions, for simplicity, it is usually supposed to zero^47^.

11 $$f\left( {x_{L \cdot i} } \right) \sim GP\left( {0 \cdot k\left( {x \cdot x^{\prime} } \right)} \right)$$

From Eqs. (7) and (11) the y is achieved as:

12 $$y \sim N\left( {0 \cdot k\left( {x \cdot x^{\prime}} \right) + \sigma_{noise}^{2} I_{n} } \right)$$

Now, based on the introduced parameters:

13 $$\left[ {\begin{array}{*{20}c} {\to _{{f_{L} }} } \\ {\to _{{f_{T} }} } \\ \end{array} } \right] \sim N\left( {0 \cdot \left[ { \begin{array}{*{20}c} {k\left( {x_{L} \cdot x_{L} } \right)} & {k\left( {x_{L} \cdot x_{T} } \right)} \\ {k\left( {x_{T} \cdot x_{L} } \right)} & {k\left( {x_{T} \cdot x_{T} } \right)} \\ \end{array} } \right]} \right)$$

14 $$\left[ {\begin{array}{*{20}c} {\to _{{\varepsilon_{L} }} } \\ {\to _{{\varepsilon_{T} }} } \\ \end{array} } \right] \sim N\left( {0 \cdot \left[ {\begin{array}{*{20}l} {\sigma_{noise}^{2} I_{n} } \hfill & 0 \hfill \\ 0 \hfill & {\sigma_{noise}^{2} I_{n} } \hfill \\ \end{array} } \right]} \right)$$

By summation of these two equations, the Gaussian expression is derived:

15 $$\left[ {\begin{array}{*{20}c} {\to _{{y_{L} }} } \\ {\to _{{y_{T} }} } \\ \end{array} } \right] \sim N\left( {0 \cdot \left[ {\begin{array}{*{20}l} {k\left( {x_{L} \cdot x_{L} } \right) + \sigma_{noise}^{2} I_{n} } \hfill & {k\left( {x_{L} \cdot x_{T} } \right)} \hfill \\ {k\left( {x_{T} \cdot x_{L} } \right)} \hfill & {k\left( {x_{T} \cdot x_{T} } \right) + \sigma_{noise}^{2} I_{n} } \hfill \\ \end{array} } \right]} \right)$$

To obtain the *y*_*T*_ distribution, the conditioning rule of Gaussians can be used:

16 $$\left( {y_{T} |y_{L} } \right)\sim N\left( {\mu_{T} \cdot {\Sigma }_{T} } \right)$$

17 $${\Sigma }_{T} = k\left( {x_{T} \cdot x_{T} } \right) = k\left( {x_{T} \cdot x_{T} } \right) + \sigma_{noise}^{2} I_{n} - k\left( {x_{T} \cdot x_{L} } \right)\left( {k\left( {x_{L} \cdot x_{L} } \right) + \sigma_{noise}^{2} I_{n} } \right)^{ - 1} k\left( {x_{L} \cdot x_{T} } \right)$$

18 $$\mu_{T} = m\left( {\to _{{y_{T} }} } \right) = k\left( {x_{T} \cdot x_{L} } \right)\left( {k\left( {x_{L} \cdot x_{L} } \right) + \sigma_{noise}^{2} I_{n} } \right)^{ - 1} \to _{{y_{T} }}$$

With *Σ*_*T*_ and *μ*_*T*_ as the covariance and the mean value, respectively. The core of the GPR is the kernel function which generates a covariance matrix to calculate the "distance" between two data points. Thus, various kernel functions have different calculation approaches, affecting the strength and the robustness of the final GPR model^48^. In the present study, four kernel functions of Matern, Rational quadratic, Exponential, and Squared exponential are chosen to find the most appropriate one, defined as follows

- Matern kernel function:
  19 $$k_{M} \left( {x \cdot x^{\prime}} \right) = \sigma^{2} \frac{{2^{1 - v} }}{{{\Gamma }\left( v \right)}}\left( {\sqrt {2v} \frac{{x - x^{\prime} }}{\ell }} \right)^{v} K_{v} \left( {\sqrt {2v} \frac{{x - x^{\prime} }}{\ell }} \right)$$
- Rational quadratic kernel function:
  20 $$k_{RQ} \left( {x \cdot x^{\prime} } \right) = \sigma^{2} \left( {1 + \frac{{x - x^{^{\prime} 2} }}{2a\ell }} \right)^{ - a}$$
- Exponential kernel function:
  21 $$k_{E} \left( {x \cdot x^{\prime} } \right) = \sigma^{2} exp\left( { - \frac{{x - x^{\prime} }}{\ell }} \right)$$
- Squared Exponential kernel function:
  22 $$k_{SE} \left( {x \cdot x^{\prime} } \right) = \sigma^{2} exp\left( { - \frac{{x - x^{^{\prime} 2} }}{{\ell^{2} }}} \right)$$

In these equations, ℓ, σ, σ^2^, and α > 0 indicate the length scale, the amplitude, the variance, and scale-mixture, respectively. Also, *v*, K_v_, and Γ represent a positive parameter, the modified Bessel function, and the gamma function, respectively.

In the present study, we developed GPR models based on four kernel functions in MATLAB software version 2018 and compared their capabilities to estimate enthalpy of absorptions.

### Data set outlier detection

**Figure 2:**
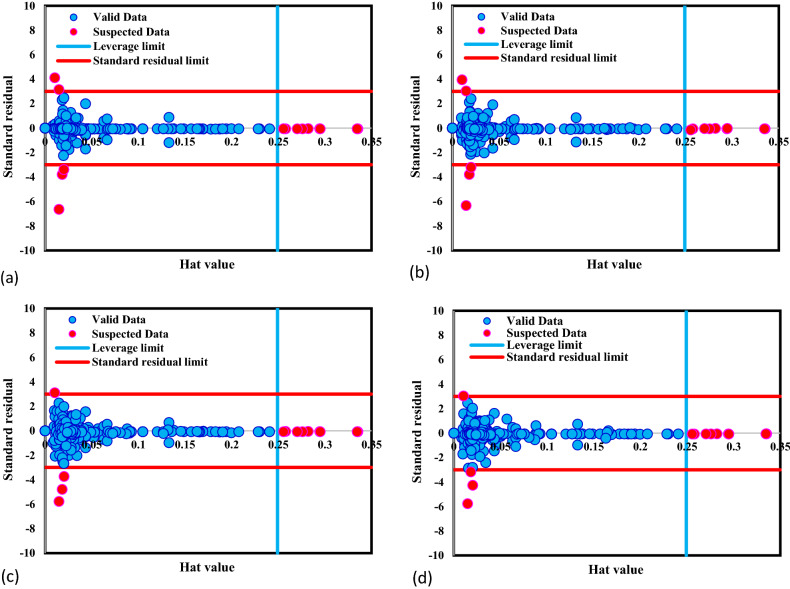
Detection of suspected data for GPR model with kernel function of (**a**) Exponential, (**b**) Matern, (**c**) Squared exponential, (**d**) Rational quadratic.

Due to the existing errors in experiments or calculation methods, some of the collected data behave differently from other data points, known as suspected data or outliers. Having these data in the data bank leads to improper anticipation for the established models. Accordingly, the presence of the suspected data in the data bank should be investigated to advance the quality of the collected data bank. For this purpose, the Leverage method is used, which defines the Hat matrix and critical leverage limit as follows:

23 $$H = U\left( {U^{T} U} \right)^{ - 1} U^{T}$$

24 $$H^{*} = \frac{{3\left( {j + 1} \right)}}{i}$$

where *U* is a matrix with the i*j dimension, and i and j are the number of parameters and the training data, respectively. To assess the quality of the collected data bank, William's plot concept is used, through which standardized residuals are portrayed versus hat values. According to the method, the data from the reliable zone, the confined area between standardized residuals of [− 3, 3] and [0, critical leverage limit], are suspected data. In the present work, as shown in Fig. 2, most data is in the reliable area. In detail, for all the developed GPR models, only 14 or 13 data points out of 314 data (about 4%) are out of the reliable zone, confirming the collected data set is appropriate for training and testing.

## Results and discussion

### Sensitivity analysis

**Figure 3:**
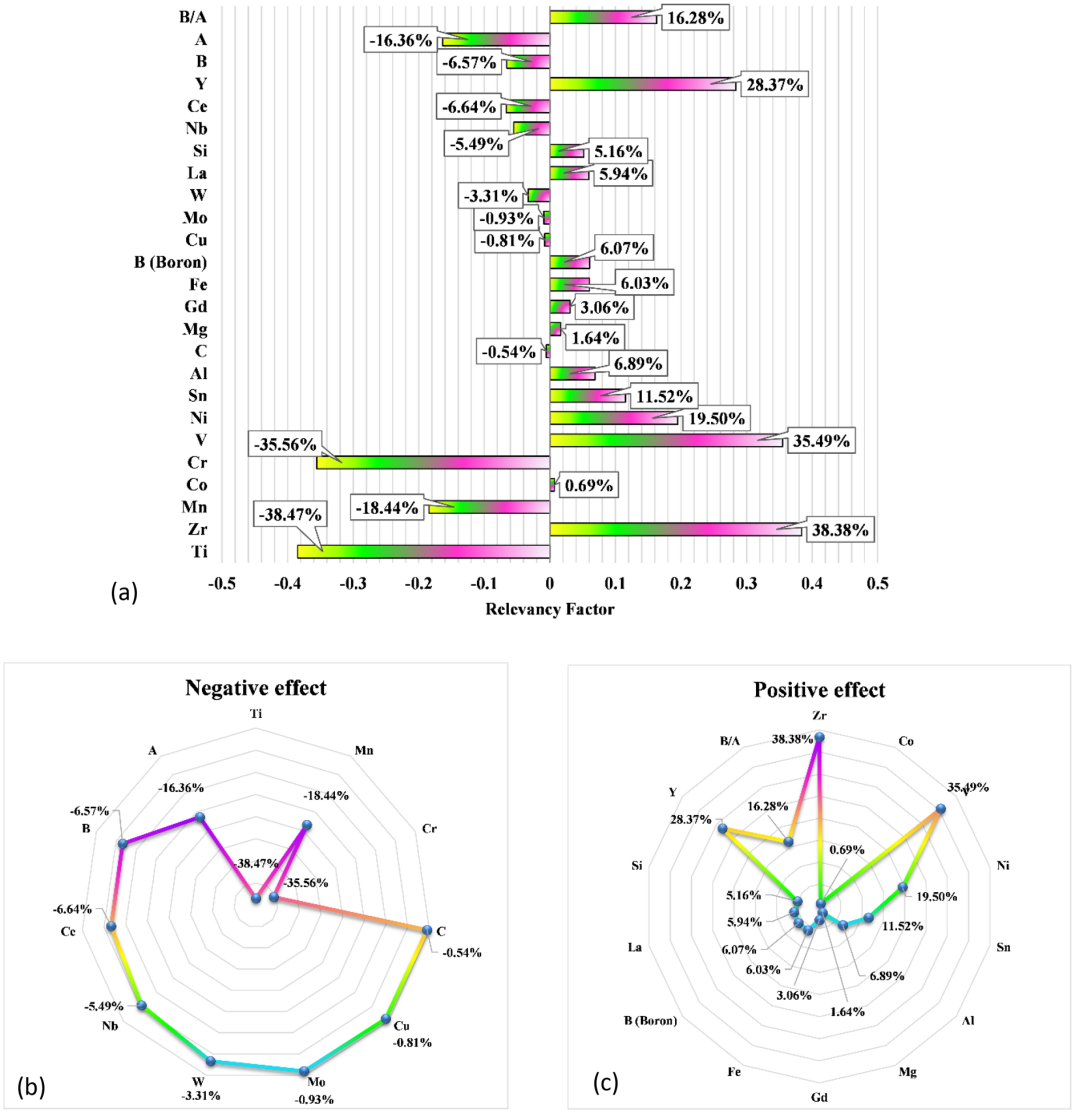
Sensitivity analysis of the input variables for ΔH absorption of hydrogen on metal hydrides.

In order to determine the effect of each element on the absorption enthalpy, an analysis of sensitivity is implemented. The relevancy factor, the metric which implies how much a parameter is effective, is derived from the following expression:

25 $$r = \frac{{\mathop \sum \nolimits_{i = 1}^{n} \left( {X_{k.i} - \overline{X}_{k} } \right)\left( {Y_{i} - \overline{Y}} \right)}}{{\sqrt {\mathop \sum \nolimits_{i = 1}^{n} \left( {X_{k.i} - \overline{X}_{k} } \right)^{2} \mathop \sum \nolimits_{i = 1}^{n} \left( {Y_{i} - \overline{Y}} \right)^{2} } }}$$

where $X_{k.i}$ and $Y_{i}$ represent the 'k' th input and 'i' th output, while the average values of input and outputs are denoted by ${\overline{X} }_{k}$ and $\overline{Y }$, respectively. The input parameter with a larger r means a greater effect on the outcome. The positive sign indicates the parameter affects the output positively and vice versa for negative signs. According to the sensitivity analysis (Fig. 3), Ti and Zr are the most effective elements in the ΔH absorption of hydrogen, with the relevancy factor of − 38.47% and 38.38%, respectively. The opposite sign of these elements is because of their interchange in A site. In other words, when Ti increases, the amount of Zr automatically decreases and vice versa. This result was expected because, as discussed, element A (here are Ti and Zr) in AB_2_ structures is the hydride forming element, significantly affecting the hydrogen adsorption energy of the alloy^49^. Among the rest of the metals placed in the B site of the AB_2_ structures, Cr and V are the most influential input elements, while C and Co have the minimum effect on the alloys-hydrogen absorption ΔH. It can be related to their abundance in the collected dataset. Indeed, Cr, Mn, and V have been extensively applied in this research area, while other metals were often used as dopants/modifiers.

### Modeling results and validation

The statistical parameters and the graphical comparison figures are presented to evaluate the developed model performance in the hydrogen absorption ΔH prediction. The statistical parameters are calculated and listed in 1 for the train, test, and overall dataset. In the training phase, the R^2^ values of 0.976, 0.976, 0.95, and 0.966 were obtained for established GPR-Exponential, GPR-Matern, GPR-Squared Exponential, and GPR-Rational Quadratic models, respectively. Considering their low amount of MRE, MSE, RMSE, and STD, especially for the GPR-Exponential model, confirms that all the GPR models were trained with enough preciseness. They were used to predict new (testing) data to examine the robustness of the models. Based on Table 1, all the developed models showed their acceptable capability in the ΔH prediction. The GPR-Exponential is slightly more accurate among all models with R^2^ = 0.969, MRE = 2.291%, MSE = 3.909, RMSE = 2.501, and STD = 1.878.

**Table 1:** The calculated statistical parameters of proposed GPR models.

Model	Group	R^2^	MRE (%)	MSE	RMSE	STD
GPR (Exponential)	Train data	0.976	2.303	3.130494859	1.7693	1.6724
	Test data	0.938	2.253	6.25850367	2.5017	2.4030
	Total data	0.969	2.291	3.909998652	2.5017	1.8782
GPR (Matern)	Train data	0.976	2.646	3.409763115	1.8466	1.7033
	Test data	0.903	3.895	7.025170203	2.6505	2.4049
	Total data	0.965	2.957	4.310727182	2.6505	1.9072
GPR (Squared Exponential)	Train data	0.955	4.136	4.856165672	2.2037	1.8525
	Test data	0.940	5.262	10.34689408	3.2167	2.9355
	Total data	0.950	4.416	6.224462209	3.2167	2.1691
GPR (Rational Quadratic)	Train data	0.966	3.711	4.844857898	2.2011	1.9656
	Test data	0.906	4.588	6.788354588	2.6054	2.2364
	Total data	0.957	3.929	5.329179756	2.6054	2.0388

**Figure 4:**
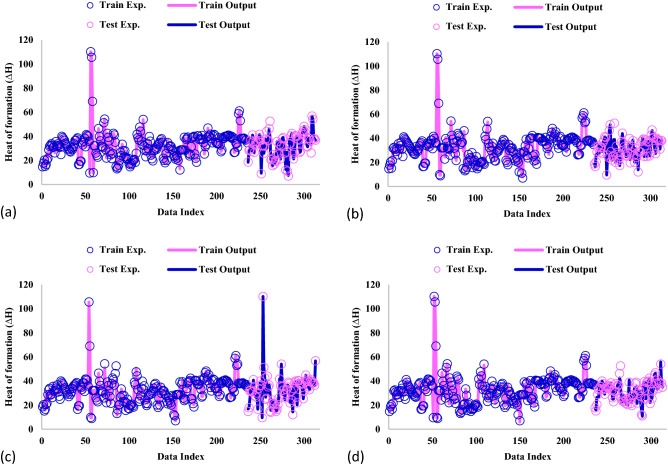
Comparison of actual and anticipated data for GPR model containing kernel function of (**a**) Exponential, (**b**) Matern, (**c**) Squared exponential, (**d**) Rational quadratic.

The simultaneous comparison between the experimental and anticipated amounts of hydrogen absorption ΔH for all the models is illustrated in Fig. 4. It is clear that all the proposed GPR models are predicted in excellent agreement with the actual values of ΔH through which the prediction lines cover the data points accurately.

**Figure 5:**
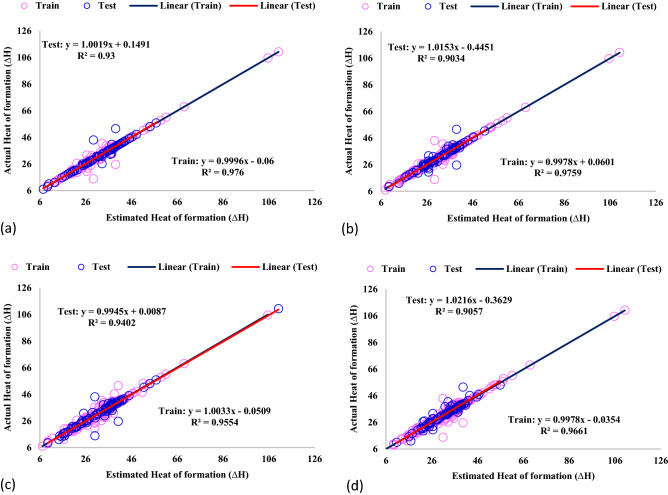
Cross plots for GPR model with kernel function of (**a**) Exponential), (**b**) Matern, (**c**) Squared exponential, (**d**) Rational quadratic.

The cross plots for all the GPR models are depicted in Fig. 5. In these graphs, the bisector line of the first quarter is the accuracy merit; the closer data to this line, the more precise model is developed. As shown in Fig. 5, the data are placed very close to the bisector line, and their respective linear fitting equations are almost the same as the merit line (slope of unit with R^2^ greater than 0.9). Thus, the established GPR models could anticipate the ΔH very well.

**Figure 6:**
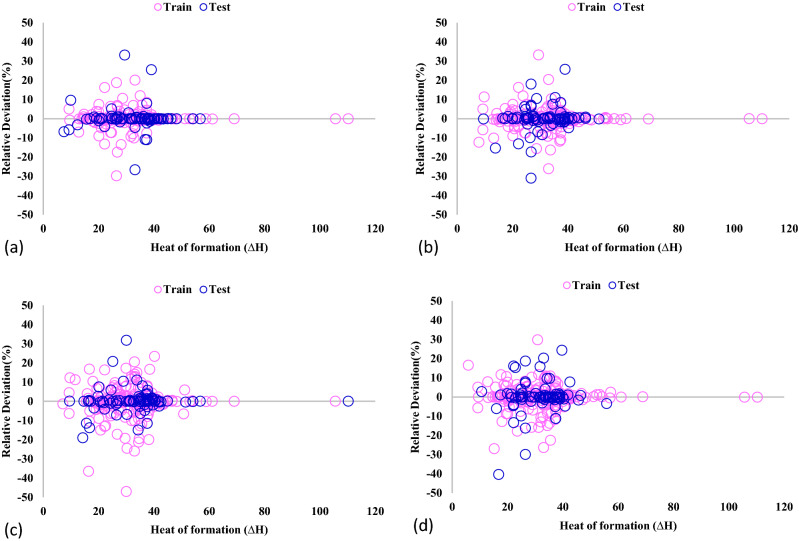
Comparison of actual and anticipated data for GPR model containing kernel function of (**a**) Exponential, (**b**) Matern, (**c**) Squared exponential, (**d**) Rational quadratic.

For more assessment of the GPR models' results, the relative deviation between the actual hydrogen absorption and the predicted ones is calculated and illustrated in Fig. 6. In each of the developed GPR models, most of the calculated absolute deviation data points are smaller than 10%. Also, the GPR model with Exponential kernel function has the minimum mean relative error of 2.291% compared to GPR-Matern (2.957%), GPR-Square exponential (4.416%), and GPR-Rational quadratic (3.929%).

## Conclusion

In order to anticipate the hydrogen absorption ΔH on the AB_2_ alloys, a machine learning approach of Gaussian process regression (GPR) with four different kernel functions (Exponential, Matern, Squared exponential, and Rational quadratic) was assessed. The 22 different alloying elements were used as the input. All the developed GPR models performed very well. Among them is the GPR-Exponential model with a little more excellence than others, with R2, MRE, MSE, RMSE, and STD of 0.969, 2.291%, and 3.909, 2.501, and 1.878, respectively, chosen as the best one. According to the sensitivity analysis, the Ti and Zr elements, along with V and Cr, contribute the most to the change of hydrogen absorption ΔH. The results of the presented work could provide the researchers and scientists with a perspective to choose the appropriate elements for AB_2_ alloys for hydrogen storage.

## Supplementary Information

Supplementary Information.

## Data Availability

All data generated or analysed during this study are included in this published article [and its supplementary information files].
